# Major pathologic response predicts survival in resectable stage IIIA non-small cell lung cancer after neoadjuvant therapy

**DOI:** 10.1093/icvts/ivae213

**Published:** 2024-12-16

**Authors:** Shihong Zhou, Ying Zhang, Ziheng Wu, Pinghui Xia, Tianyu He, Jinlin Cao, Wang Lv, Jian Hu

**Affiliations:** Department of Thoracic Surgery, the First Affiliated Hospital, Zhejiang University School of Medicine, Hangzhou, China; Department of Radiation Oncology, Shanghai Pulmonary Hospital, Tongji University Medical School Cancer Institute, Tongji University School of Medicine, Shanghai, China; Department of Thoracic Surgery, the First Affiliated Hospital, Zhejiang University School of Medicine, Hangzhou, China; Department of Thoracic Surgery, the First Affiliated Hospital, Zhejiang University School of Medicine, Hangzhou, China; Department of Thoracic Surgery, the First Affiliated Hospital, Zhejiang University School of Medicine, Hangzhou, China; Department of Thoracic Surgery, the First Affiliated Hospital, Zhejiang University School of Medicine, Hangzhou, China; Department of Thoracic Surgery, the First Affiliated Hospital, Zhejiang University School of Medicine, Hangzhou, China; Department of Thoracic Surgery, the First Affiliated Hospital, Zhejiang University School of Medicine, Hangzhou, China; Key Laboratory of Clinical Evaluation Technology for Medical Device of Zhejiang Province, Hangzhou, China

**Keywords:** major pathologic response, long-term prognosis, non-small cell lung cancer, neoadjuvant chemotherapy, neoadjuvant immunochemotherapy, real-world retrospective study

## Abstract

**OBJECTIVES:**

Major pathologic response is more common in survival analyses than pathological complete response. Whether major pathologic response can predict survival of patients with resectable stage IIIA non-small cell lung cancer and whether neoadjuvant chemotherapy or immunochemotherapy affect the prognosis of patients remains questionable.

**METHODS:**

Patients with resectable stage IIIA non-small cell lung cancer receiving neoadjuvant chemotherapy (≥2 cycles) with/without immunotherapy were enrolled and divided into two groups according to pathological response. Comparison between the two groups was through chi-square test. Univariate Cox regression analysis and log-rank test were made to identify predictive factors of overall survival and disease-free survival. Kaplan–Meier survival curves were constructed to evaluate the prognostic impact of these factors.

**RESULTS:**

Totally, 38 patients were enrolled. Significant difference was observed in overall survival (*P* = 0.005) and disease-free survival (*P* = 0.007) between patients with/without major pathologic response. For patients failing to reach major pathologic response, those who underwent ≥2 cycles of neoadjuvant therapy exhibited improved outcomes in overall survival (*P* = 0.021) and disease-free survival (*P* = 0.046). Notably, within this subgroup, patients receiving ≥ 2 cycles of neoadjuvant immunochemotherapy showed a trend towards better overall survival (*P* = 0.076) and disease-free survival (*P* = 0.062).

**CONCLUSIONS:**

Major pathologic response can predict survival of patients with resectable stage IIIA non-small cell lung cancer. For patients potentially not achieving major pathologic response after two cycles of neoadjuvant therapy, extended cycles of feasible neoadjuvant therapy are advisable for survival benefits.

## Graphical abstract

**Figure:**
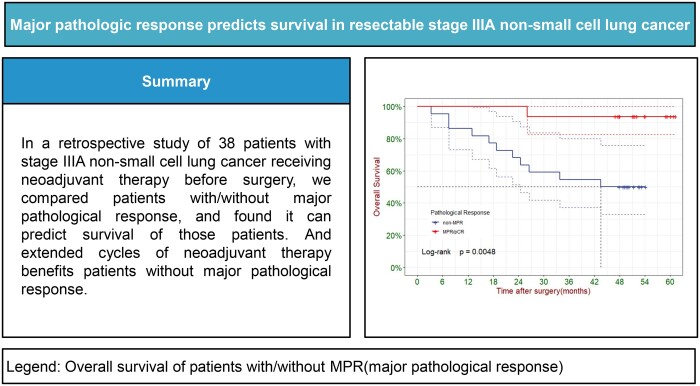

## INTRODUCTION

Non-small cell lung cancer (NSCLC) is the leading subtype, accounting for around 80%–85%, of lung cancer [1]. Nowadays, surgery remains the mainstay of treatment for early-stage NSCLC [2]. Over the past few decades, for patients with locally advanced NSCLC, the advent of preoperative neoadjuvant therapy increased the opportunities of surgery and improved the rate of resection [3].

Neoadjuvant therapy has a lot of advantages, including improving the patient’s tolerance to surgery, tumour downstaging, providing an earlier chance to eradicate micro-metastases, and more rapid assessment of therapeutic effect both before and during surgery [4–6]. Currently, neoadjuvant therapy for NSCLC included neoadjuvant chemotherapy (NACT), neoadjuvant immunotherapy and neoadjuvant immunochemotherapy (NAICT) [7–9]. Immune checkpoint inhibitors (ICIs), which are targeting the programmed cell death protein 1 (PD-1)/programmed death ligand 1 pathway, with/without chemotherapy, have been developed for treatment of metastatic NSCLC [2, 10]. Several trials further explored the application of ICIs as monotherapy or combining chemotherapy in perioperative setting for resectable NSCLC. Based on CheckMate-816 trial, nivolumab became the first immunotherapy drugs approved for neoadjuvant therapy [11]. Recent results from the AEGEAN trial and the KEYNOTE-617 trial have demonstrated the benefit of durvalumab or pembrolizumab, respectively, significantly improving disease-free survival (DFS), major pathological response (MPR) and pathological complete response (pCR) in resectable NSCLC [12, 13].

For NSCLC, MPR rate ranges from 19% to 45% in NACT, and fluctuates between 33% and 83% in NAICT [14]. Although overall survival (OS) is considered the gold-standard outcome measure in oncological studies, longer follow-up may delay the translation of potentially effective treatment into clinical practice. Some research studies began advocating MPR as a candidate surrogate end-point to rapidly assess the clinical efficacy of NACT without immunotherapy [15, 16]. What’s more, the correlation between MPR rate and cycles of neoadjuvant therapy for locally advanced NSCLC patients has been reported [17, 18]. However, whether cycles of NACT or NAICT affect their prognosis remains questionable.

In this study, we retrospectively analysed 38 consecutive patients with resectable stage IIIA NSCLC receiving NACT with/without PD-1 inhibitors followed by radical surgery and made a long-term follow-up to investigate whether MPR could predict prognosis. In addition, whether NACT or NAICT affects the prognosis of patients were also discussed.

## MATERIALS AND METHODS

### Patients

This study conformed to the provisions of the Declaration of Helsinki (revised in 2013). Clinical Research Ethics Committee of the First Affiliated Hospital, Zhejiang University School of Medicine, gave ethical approval for this study (IIT20230566A), and written informed consent was waived.

Patients with resectable stage IIIA NSCLC who received NACT with/without PD-1 inhibitors, totally ≥2 cycles, at the First Affiliated Hospital, Zhejiang University School of Medicine, from September 2019 to December 2020 were enrolled. Inclusion and exclusion criteria of this study, and methods of clinical staging at baseline were shown in the Supplementary Material. Malignant tumours were staged according to the 8th edition TNM staging system.

### Drug treatment

Patients were administered 2–8 cycles of NACT with/without PD-1 inhibitors, and the specific scheme was determined according to pathological type of tumours (Supplementary Material). The immunotherapy agents included five PD-1 inhibitors (intravenous drip on the first day and 21 days as a cycle), which were pembrolizumab (2 mg/kg), nivolumab (3 mg/kg), sintilimab (200 mg), camrelizumab (200 mg) and tislelizumab (200 mg). The selection of different immunotherapy agents was determined by the approved indications, price of the drug and financial capacity of patients. Regular haematological and imaging examinations were performed during treatment.

After neoadjuvant therapy, clinical restaging (ycStage) was evaluated by two clinical surgeons, and radical surgery was proceeded 2–4 weeks later (Supplementary Material). Furthermore, the use of ICIs was consistent across both NAICT and adjuvant therapy phases for patients in our study.

### Evaluation of survival outcome

Assessment of tumour response to neoadjuvant therapy were through tumour changes in size on preoperative CT or PET-CT imaging by chief thoracic surgeons, according to Response Evaluation Criteria in Solid Tumors 1.1(RECIST1.1) [19]. Clinical response indicators were complete response, partial response, disease progression and objective response rate. After first two cycles of neoadjuvant therapy, patients were evaluated for possibility of reaching MPR on postoperative pathological response by imaging methods. Whether to prolong the cycle of neoadjuvant therapy or to change the type of neoadjuvant therapy was considered by clinical surgeons. Finally, ypStage of resected tumour and pathologic responses to neoadjuvant therapy were confirmed by two experienced pathologists, re-reviewing pathologic slides. MPR was defined as ≥90% necrosis of tumour, and pCR was defined as tumour regression with no residuals pathologically. After neoadjuvant therapy, if both above conditions were not achieved, patient would be classified into non-MPR.

### Follow-up

Follow-up time (months) was determined from date of surgery until date of death due to any cause or end of follow-up (October 23, 2024) whichever occurred first. Patients were followed up every 1–3 months in the first 2 years after surgery, and every 6 months thereafter. Oncological follow-up consisted of chest CT and ultrasonography of liver, gallbladder, spleen, pancreas and bilateral adrenal glands at every regular outpatient visit. Bronchoscopy was performed annually after surgery. Primary end-points were OS and DFS. OS was defined from the date of surgery to the time of death from any cause or the last follow-up. DFS was defined as the period from the surgery day to the time of first recurrence, death from any cause or the last follow-up. Complete follow-up information was available for all patients.

### Statistical analysis

Comparisons between MPR/pCR and non-MPR groups were analysed using chi-square test for categorical variables. We measured the predictive effect of clinicopathological characteristics, including MPR/pCR vs. non-MPR, on prognosis using univariable Cox analysis and log-rank test. Furthermore, subgroup analysis in non-MPR group was performed to see predictive factors of neoadjuvant therapy with improved prognosis. Kaplan–Meier curves for survival analysis were plotted and compared using a log-rank test. Statistical analyses were performed with R software version 4.1.0. All tests were 2-sided, and *P*-value < 0.05 were considered significant. All data were retained to three decimal places.

## RESULTS

### Patients’ characteristics

**Figure 1:**
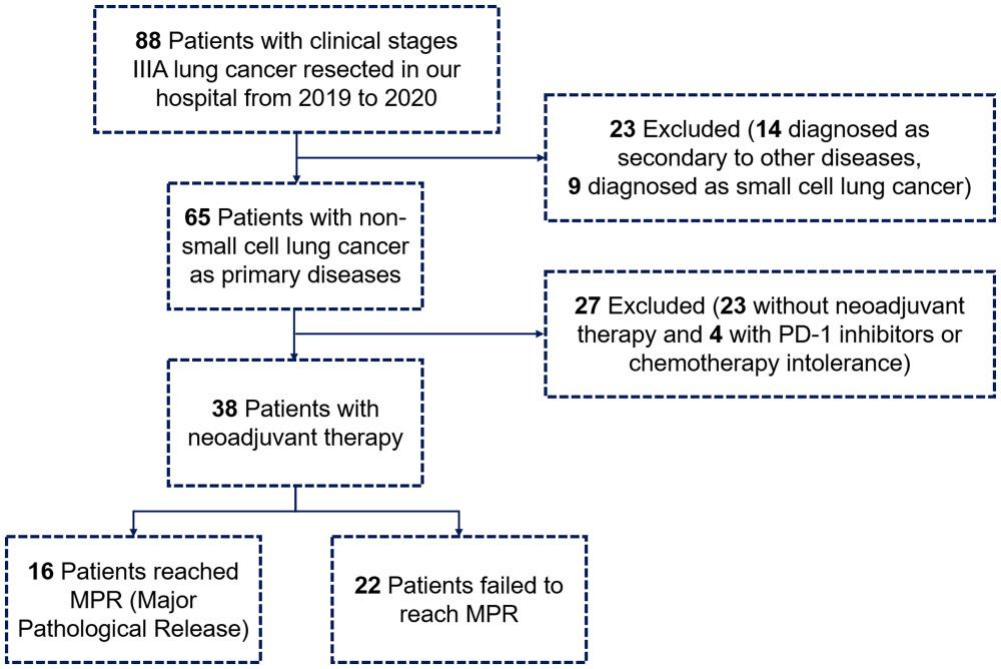
The flow diagram of this study.

From September 2019 to December 2020, 38 eligible patients were enrolled in this study (NACT+NAICT/NAICT, *n* = 32, 84.2%; NACT, *n* = 6, 15.8%), among whom 16 (42.1%) reached MPR/pCR, including 12 (31.6%) reached pCR, and 22 (57.9%) failed. Details of the flow diagram was shown in Fig. 1. Patient characteristics were listed in Table 1. For patients receiving NACT+NAICT/NAICT, MPR rate is 46.9% and pCR rate is 25.0%. For those receiving NACT alone, MPR rate is 16.7% and pCR rate is 0.0%. For patients receiving two cycles of neoadjuvant therapy, MPR/pCR rate is 44.4%. For those who receiving >2 cycles of neoadjuvant therapy, MPR/pCR rate is 40.0%. The objective response rate of whole cohort was 71.1%, with 27 patients reaching complete response or partial response clinically. According to the ycStage after neoadjuvant therapy, 20 patients (52.6%) down-staged to clinical IA, 3 patients (7.9%) down-staged to clinical IB, 2 patients (5.3%) down-staged to clinical IIA, while 13 patients (34.2%) remained clinical IIIA. More details of enrolled patients could be seen in Supplementary Material, Table S1.

**Table 1:** Clinicopathological characteristics

Variables	Total (*n* = 38)	MPR/pCR (*n* = 16)	Non-MPR (*n* = 22)	*P*-value
Age				0.902
>65 years	23	9	14	
≤65 years	15	7	8	
Gender				0.205
Male	34	16	18	
Female	4	0	4	
ECOG-PS				0.701
=1	5	3	2	
=0	33	13	20	
Type of neoadjuvant therapy				0.355
NACT+NAICT/NAICT	32	15	17	
NACT	6	1	5	
Cycle of neoadjuvant therapy				1.000
>2	20	8	12	
=2	18	8	10	
Cycle of NAICT				1.000
>2	14	6	8	
≤2	24	10	14	
Clinical response				0.274
CR+PR	27	13	14	
SD+PD	11	3	8	
ycStage				0.752
III	12	6	6	
I/II	26	10	16	
Surgery approach				0.186
Thoracotomy	9	6	3	
VATS/RATS	19	10	9	
Histology				0.071.
LUAD	12	2	10	
LUSC	26	14	12	
Resection margin				0.773
Non-R0	3	2	1	
R0	35	14	21	
Reoperation after surgery				1.000
Yes	2	1	1	
No	36	15	21	
Pathological stage				0.038*
ypStage III/IV	7	0	7	
ypStage I/II	31	16	15	
Cycle of adjuvant therapy				0.076
>6	14	9	5	
≤6	24	7	17	
Death				0.012*
Yes	12	1	11	
No	26	15	11	
Recurrence				0.016*
Yes	17	3	14	
No	21	13	8	

*Factors with P-value<0.05 were considered significant and the P-value was shown in bold.

Between these two groups, type of neoadjuvant therapy (NACT+NAICT/NAICT vs. NACT), cycle of neoadjuvant therapy, cycle of NAICT, clinical response and ycStage showed no significant difference after chi-square test (Table 1). However, ypStage (*P* = 0.038), death (*P* = 0.012) and recurrence (*P* = 0.016) was significantly different between MPR/pCR and non-MPR groups. Patients in MPR/pCR group exhibited a higher incidence of pathological downstaging, and all achieved ypStage I/II after surgery. Patients in MPR/pCR group showed a better survival rate and a reduced recurrence rate.

### Overall survival and disease-free survival analysis for whole cohort

The median follow-up time was 49.4 months, with a maximum of 61.3 months in whole cohort. In total, 1 (6.3%) of 16 MPR/pCR patients and 11 (50.0%) of 22 non-MPR patients died until the last follow-up in OS analysis. In DFS analysis, 3 (18.8%) MPR/pCR patients and 14 (63.6%) non-MPR patients experienced recurrence by the last follow-up.

In OS analysis, pathologic response (Cox *P* = 0.024, log-rank *P* = 0.005) and ypStage (Cox *P* = 0.017, log-rank *P* = 0.010) were found significantly associated with OS in both univariate Cox analysis and log-rank test (Table 2). Other factors including type of neoadjuvant therapy, cycle of neoadjuvant therapy and cycle of NAICT showed no statistical significance.

**Table 2:** Univariable Cox analysis and log-rank test for overall survival and disease-free survival of the whole cohort

Variables	Univariate Cox analysis			Log-rank test		
	HR	95% CI	*P*-value	HR	95% CI	*P*-value
Overall survival						
Pathological response:	1.094	0.012–0.729	0.024*	0.095	0.031–0.295	0.005*
MPR/pCR vs. Non-MPR						
Age > 65 years	1.011	0.321–3.188	0.985	1.011	0.321–3.182	0.985
Male vs. Female	0.509	0.111–2.326	0.384	0.510	0.072–3.592	0.375
ECOG-PS	0.589	0.076–4.566	0.613	0.589	0.112–3.108	0.608
NACT+NAICT/NAICT vs. NACT	0.855	0.187–3.906	0.840	0.855	0.172–4.245	0.840
Cycle of neoadjuvant therapy > 2	0.511	0.162–1.563	0.231	0.496	0.157–1.568	0.221
Cycle of NAICT > 2	0.487	0.132–1.798	0.280	0.487	0.154–1.541	0.270
Clinical Response:	1.237	0.393–3.897	0.717	1.236	0.382–4.001	0.717
CR+PR vs. SD+PD						
ycStage III vs. I/II	0.978	0.294–3.250	0.970	0.978	0.296–3.232	0.970
VATS/RATS vs. Thoracotomy	0.951	0.257–3.516	0.940	0.951	0.253–3.573	0.940
LUAD vs. LUSC	1.099	0.331–3.650	0.878	1.099	0.324–3.723	0.878
R0 vs. Non-R0	1.133	0.146–8.785	0.905	1.133	0.162–7.920	0.905
Reoperation	1.617	0.208–12.570	0.646	1.614	0.130–20.035	0.643
ypStage III/IV vs. I/II	4.107	1.289–13.090	0.017*	4.027	0.828–10.584	0.010*
Cycle of adjuvant therapy > 6	0.783	0.236–2.600	0.689	0.783	0.245–2.497	0.688
Disease-free survival						
Pathological response:	0.212	0.061–0.741	0.015*	0.214	0.083–0.553	0.007*
MPR/pCR vs. Non-MPR						
Age > 65 years	0.917	0.349–2.411	0.860	0.914	0.345–2.422	0.855
Male vs. Female	1.085	0.248–4.750	0.914	1.082	0.259–4.531	0.916
ECOG-PS	0.917	0.349–2.411	0.860	0.367	0.095–1.412	0.308
NACT+NAICT/NAICT vs. NACT	0.572	0.186–1.759	0.330	0.572	0.151–2.168	0.321
Cycle of neoadjuvant therapy > 2	0.648	0.250–1.681	0.372	0.647	0.247–1.693	0.364
Cycle of NAICT > 2	0.629	0.222–1.787	0.384	0.627	0.237–1.654	0.374
Clinical response:	0.933	0.345–2.525	0.891	0.928	0.347–2.485	0.883
CR+PR vs. SD+PD						
ycStage III vs. I/II	1.690	0.641–4.456	0.289	1.673	0.590–4.744	0.289
VATS/RATS vs. Thoracotomy	1.411	0.405–4.911	0.589	1.414	0.459–4.357	0.583
LUAD vs. LUSC	1.577	0.598–4.158	0.357	1.576	0.563–4.416	0.349
R0 vs. Non-R0	0.773	0.176–3.386	0.732	0.771	0.150–3.956	0.729
Reoperation	2.963	0.666–13.180	0.154	2.858	0.286–28.609	0.141
ypStage III/IV vs. I/II	5.068	1.817–14.130	0.002*	4.747	0.994–22.670	0.001*
Cycle of adjuvant therapy > 6	0.625	0.220–1.775	0.377	0.625	0.237–1.649	0.371

*Factors with P-value<0.05 were considered significant and the P-value was shown in bold.

Similar results were found in DFS analysis. Both univariate Cox analysis and log-rank test indicated that pathologic response (Cox *P* = 0.015, log-rank *P* = 0.007) and ypStage (Cox *P* = 0.002, log-rank *P* = 0.001) were significant factors, while other factors including type of neoadjuvant therapy, cycle of neoadjuvant therapy and cycle of NAICT demonstrated no significant association with DFS (Table 2).

**Figure 2:**
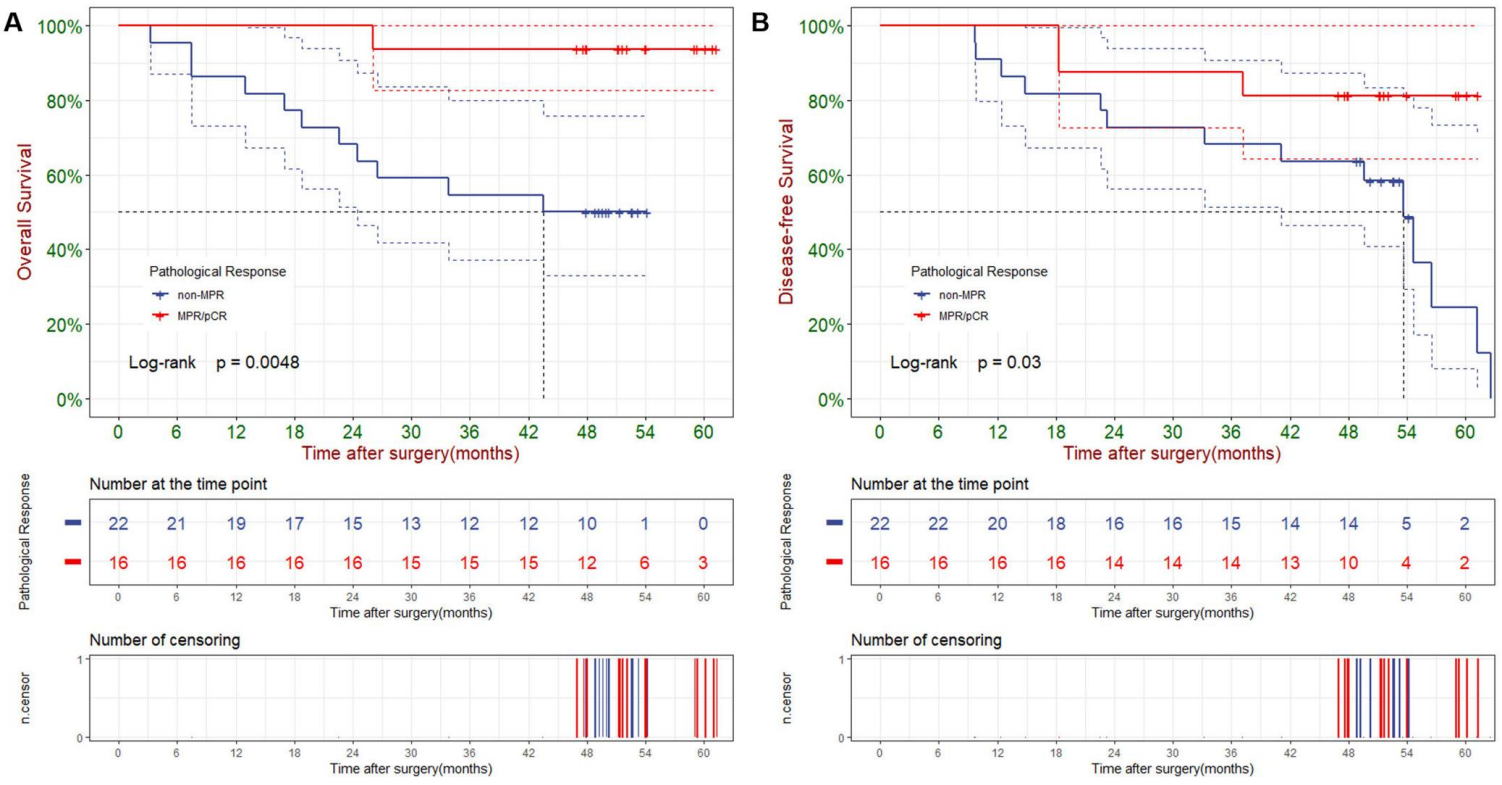
Overall survival (**A**) and disease-free survival (**B**) of all patients according to their pathologic response.

In Kaplan–Meier survival analysis, pathologic response showed significant correlation with both OS and DFS (Fig. 2A and B). Median OS was 43.5 months in non-MPR group, which was not reached in MPR/pCR group (3-year OS rate: 93.8% vs. 54.5%, *P* = 0.005). Median DFS was 37.2 months in non-MPR group, which was also not reached in MPR/pCR group (3-year DFS rate: 87.5% vs. 50.0%, *P* = 0.007). Cumulative event and cumulative hazard curves of death among all patients according to their pathological response were shown in Supplementary Material, Fig. S1. With regard to cycle of neoadjuvant therapy, there was no significant difference between cycle = 2 and cycle > 2 in either OS analysis (*P* = 0.221) or DFS analysis (*P* = 0.364) (Supplementary Material, Fig. S2).

### Overall survival and disease-free survival analysis for non-major pathologic response group

Since pathological response (MPR/pCR vs. non-MPR) was a significant prognostic factor for OS and DFS in whole cohort, we conducted a further investigation to identify significant prognostic factors within non-MPR group.

In OS analysis of non-MPR group, univariate Cox analysis found that only cycle of neoadjuvant therapy was significant (*P* = 0.031) (Table 3). Cycle of NAICT was found with a potential correlation associated with OS (*P* = 0.098). Moreover, log-rank test further verified the correlation between cycle of neoadjuvant therapy and OS (*P* = 0.021). Moreover, >2 cycles of NAICT also demonstrated a potential trend towards better OS in Table 3 (*P* = 0.076), although statistical significance was not reached.

**Table 3:** Univariable Cox analysis and log-rank test for overall survival and disease-free survival of non-MPR group

Variables	Univariate Cox analysis			Log-rank test		
	HR	95%CI	*P*-value	HR	95%CI	*P*-value
**Overall survival**						
Age > 65 years	0.773	0.235–2.543	0.672	0.774	0.231–2.598	0.671
Male vs. Female	0.985	0.212–4.573	0.984	0.985	0.211–4.592	0.984
ECOG-PS	1.170	0.149–9.180	0.881	1.169	0.130–10.485	0.881
NACT+NAICT/NAICT vs. NACT	1.323	0.285–6.133	0.721	1.322	0.323–5.418	0.720
Cycle of neoadjuvant therapy > 2	0.254	0.073–0.885	0.031*	0.266	0.075–0.946	0.021*
Cycle of NAICT > 2	0.273	0.059–1.269	0.098 .	0.276	0.084–0.905	0.076 .
Clinical response:	1.735	0.507–5.943	0.380	1.731	0.441–6.800	0.374
CR+PR vs. SD+PD						
ycStage III vs. I/II	0.830	0.220–3.138	0.784	0.831	0.232–2.978	0.783
VATS/RATS vs. Thoracotomy	0.614	0.132–2.859	0.535	0.616	0.100–3.786	0.531
LUAD vs. LUSC	0.589	0.172–2.019	0.400	0.591	0.181–1.927	0.395
R0 vs. Non-R0	0.649	0.083–5.086	0.680	0.650	0.055–7.674	0.678
Reoperation	18.100	0.000–Inf	0.999	0.000	0.000–0.000	0.397
ypStage III/IV vs. I/II	2.036	0.615–6.739	0.244	2.015	0.550–7.386	0.235
Cycle of adjuvant therapy > 6	1.242	0.328–4.698	0.750	1.241	0.306–5.029	0.749
**Disease-free survival**						
Age > 65 years	0.615	0.211–1.791	0.373	0.620	0.201–1.911	0.368
Male vs. Female	2.046	0.455–9.188	0.350	2.032	0.609–6.781	0.340
ECOG-PS	1.735	0.507–5.943	0.380	0.861	0.128–5.813	0.885
NACT+NAICT/NAICT vs. NACT	0.722	0.225–2.316	0.584	0.724	0.206–2.541	0.582
Cycle of neoadjuvant therapy > 2	0.351	0.120–1.027	0.056	0.362	0.118–1.106	0.046*
Cycle of NAICT > 2	0.314	0.087–1.131	0.077	0.318	0.111–0.909	0.062 .
Clinical response:	1.074	0.336–3.429	0.904	1.074	0.331–3.487	0.904
CR+PR vs. SD+PD						
ycStage III vs. I/II	1.668	0.550–5.056	0.366	1.647	0.493–5.503	0.361
VATS/RATS vs. Thoracotomy	0.668	0.148–3.010	0.600	0.671	0.118–3.829	0.597
LUAD vs. LUSC	1.065	0.372–3.044	0.907	1.064	0.373–3.035	0.907
R0 vs. Non-R0	0.851	0.110–6.582	0.877	0.853	0.096–7.539	0.877
Reoperation	1.343	0.173–10.440	0.778	1.337	0.133–13.455	0.778
ypStage III/IV vs. I/II	3.008	1.012–8.940	0.048*	2.855	0.786–10.378	0.038*
Cycle of adjuvant therapy > 6	0.798	0.222–2.870	0.729	0.799	0.240–2.659	0.729

*Factors with P-value<0.05 were considered significant and the P-value was shown in bold.

As for DFS analysis of non-MPR group, univariate Cox analysis showed that only ypStage (*P* = 0.048) was significantly associated with DFS, while cycle of neoadjuvant therapy (*P* = 0.056) and cycle of NAICT (*P* = 0.077) indicate a potential correlation with DFS, although without statistical significance (Table 3). However, in log-rank test, both cycle of neoadjuvant therapy (*P* = 0.046) and ypStage (*P* = 0.038) showed significant association with DFS, while >2 cycles of NAICT still demonstrated a trend with better DFS in Table 3 (*P* = 0.062).

**Figure 3:**
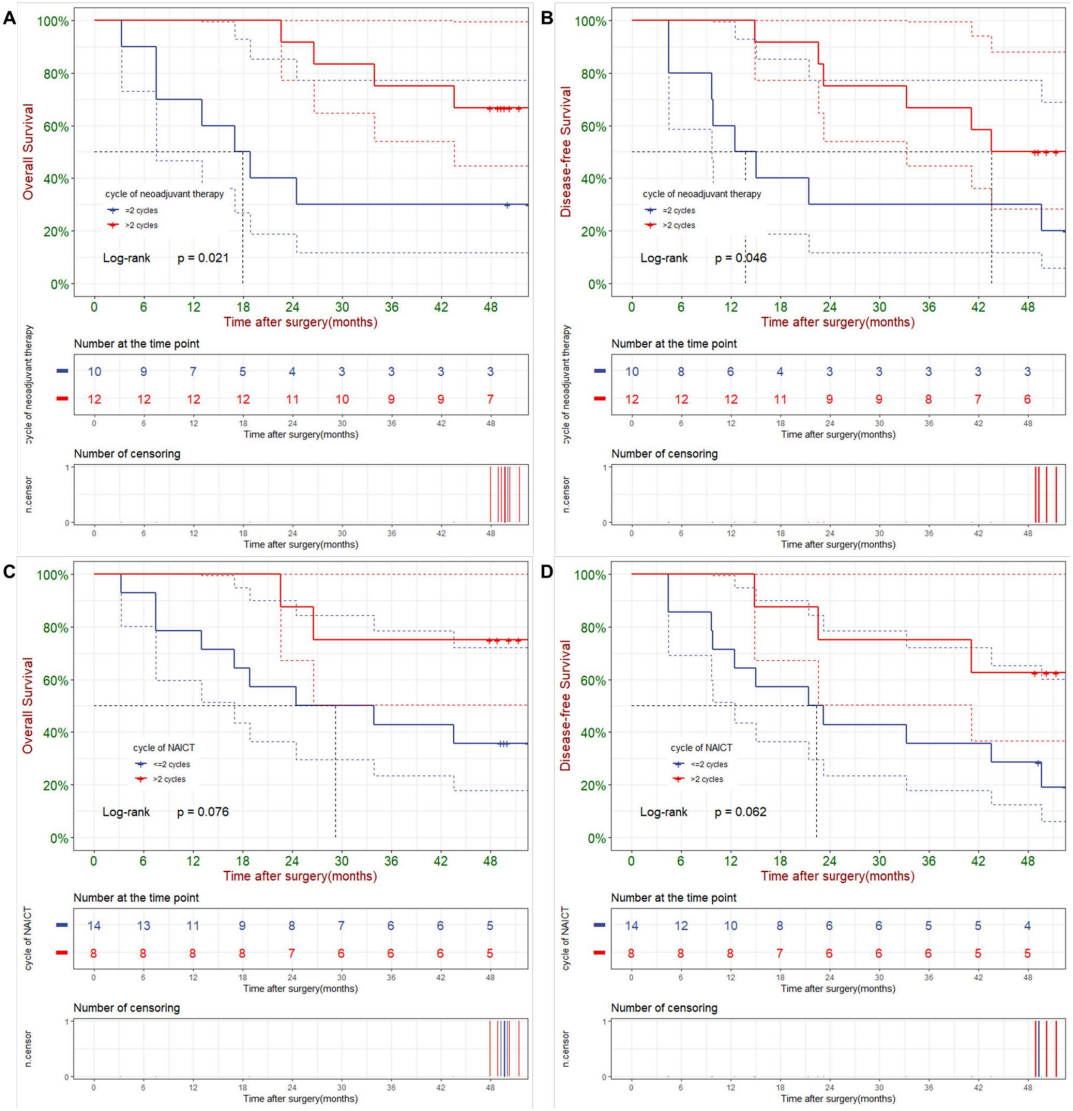
Overall survival and disease-free survival of patients in non-MPR group according to cycle of neoadjuvant therapy [(**A**) and (**B**)] and cycle of NAICT [(**C**) and (**D**)].

In Kaplan–Meier survival analysis, patients receiving >2 cycles of neoadjuvant therapy reached significantly better OS in non-MPR group (Median OS: NA vs. 17.9 months, 3-year OS rate: 75.0% vs. 30.0%, *P* = 0.021) and better DFS (Median DFS: 43.5 vs. 13.7 months, 3-year DFS rate: 66.7% vs. 30.0%, *P* = 0.046) (Fig. 3A and B). What’s more, cycle of NAICT might also influence the prognosis for non-MPR patients, and >2 cycles of NAICT showed a relatively better OS and DFS (Fig. 3C and D) (Median OS: NA vs. 29.2 months, 3-year OS rate: 75.0% vs. 42.9%, *P* = 0.076) (Median DFS: NA vs. 22.3 months, 3-year DFS rate: 75.0% vs. 35.7%, *P* = 0.062).

## DISCUSSION

Theoretically, neoadjuvant therapy offers possibility of radical resection by downstaging tumour, and opportunity of survival benefits, for patients with resectable NSCLC [2, 3]. While MPR and pCR were surrogate measures provided by neoadjuvant therapy to evaluate clinical efficacy after treatment [7]. Compared with pCR, MPR is more common in survival analysis and can save time of data accumulation and maturation [20]. To our awareness, this is the first clinical research to identify the prognostic value of MPR for patients with stage IIIA NSCLC after NACT with/without PD-1 inhibitors, and the predictive factors for patients who failed to achieve MPR.

In this research, pathological response (MPR/pCR vs. non-MPR) was found to be a significant prognostic factor for both OS and DFS. This result revealed that achieving MPR could serve as a critical threshold for predicting favourable survival outcomes, which corresponded to other previous researches [2, 15, 21–23]. What’s more, clinical response after neoadjuvant therapy, which was also an alternative prognostic predictor based mainly on the radiological response of tumour and levels of serum tumour markers, was found no significance in predicting prognosis, which is consistent with findings of Zuo *et al.* [3].

In subgroup analysis for non-MPR, our univariate Cox analysis and log-rank test identified the significant correlation between extended cycles of neoadjuvant therapy (>2), and improved OS or DFS. What’s more, cycle of NAICT (>2) was also found closely associated with both OS and DFS. These findings suggested that for patients assessed as potential non-MPR after two cycles of neoadjuvant therapy, extending cycles of neoadjuvant therapy, particularly NAICT if applicable, may be a viable option. Based on our findings, we have firstly identified predictive role of neoadjuvant therapy cycles in determining survival outcomes. Moreover, it has been reported that extending to 3–4 cycles instead of 2 cycles of NAICT may improve safety of surgery and result in a lower incidence of postoperative morbidities for NSCLC patients [17]. Zhu *et al.* found that compared with patients who received >2 cycles NAICT, patients with 2 cycles NAICT had significantly smaller diagnostic radiological tumour size and radiological tumour regression rate [18].

In clinical practice, some patients might have a relatively slow clinical response to neoadjuvant therapy, and they might strongly desire to extend cycles of neoadjuvant therapy for a better prognosis, so more cycles of neoadjuvant therapy (up to 8 cycles) might occur in this study.

In our study, MPR/pCR rate were found to be higher for patients receiving NACT combining with ICIs (in whole or in part) than those with NACT alone, although no significant difference was shown in survival. It demonstrated addition of immunotherapy to NACT could improve pathological responses to some degree. Synergistic effect of chemotherapy and ICIs, with cytotoxic chemotherapy increasing the recognition of these agents as immunotherapies, might explain the high rates of MPR [24, 25].

It is necessary to acknowledge the limitations. First, as a retrospective single-centre study, generalizability of our findings might be constrained. Second, due to nature of real-world study, potential influence of confounding factors cannot be entirely excluded. Furthermore, the small sample size could lead to bias. Therefore, to avoid even higher beta error, multivariate Cox analysis was waived, while unadjusted comparative analysis (log-rank test) was performed in this study. In addition, this study adopted five different PD-1 inhibitors as immunotherapy agents, although they are presumed to possess comparable pharmacological effects, their efficacy and safety profiles may vary in clinical practice. In future, prospective comparative trials with larger sample sizes are warranted to draw more definitive conclusions.

## CONCLUSION

MPR is a prognostic marker for predicting long-term survival in patients with resectable stage IIIA NSCLC after neoadjuvant therapy. For those patients who might potentially fail to achieve MPR after first two cycles of treatment, extending cycles of neoadjuvant therapy is recommended to improve survival outcomes.

## Supplementary Material

ivae213_Supplementary_Data

## Data Availability

The data underlying this article will be shared on reasonable request to the corresponding author.

## ABBREVIATIONS

DFS: Disease-free survival
ICI: Immune checkpoint inhibitor
MPR: Major pathologic response
NACT: Neoadjuvant chemotherapy
NAICT: Neoadjuvant immunochemotherapy
NSCLC: Non-small cell lung cancer
pCR: Pathological complete response
PD-1: Programmed cell death protein 1
