# Supplementary material for: Major pathologic response predicts survival in resectable stage IIIA non-small cell lung cancer after neoadjuvant therapy
Source: Interdiscip Cardiovasc Thorac Surg. 2024 Dec 16;40(1):ivae213. doi: 10.1093/icvts/ivae213 (PMC11681939; doi:10.1093/icvts/ivae213)
Supplement: ivae213_Supplementary_Data [file ivae213_supplementary_data.docx]

**Inclusion and exclusion criteria of this study**

Inclusion criteria:

1. NSCLC, resectable stage IIIA diagnosed by imaging and cytological examination at baseline;
2. feasible neoadjuvant therapy(≥2 cycles) for each patient;
3. patients without distant metastasis;
4. patients without radiotherapy and chemotherapy before baseline;
5. Karnofsky performance status(KPS) score ≥80;
6. patients without lung tumor operation before baseline.

Exclusion criteria:

1. patients with PD-1 inhibitors or chemotherapy intolerance;
2. patients with different ICIs used in NAICT and adjuvant therapy phases;
3. patients with other malignant tumors;
4. patients with liver, kidney, or other organ dysfunction;
5. patients with surgical contraindications;
6. patients who were lost during follow-up.

**Clinical staging methods**

Clinical staging at baseline was performed using imaging methods, such as computed tomography(CT), bone scan and/or positron emission tomography(PET-CT). Cytological examination, including fiberoptic bronchoscopy and endobronchial ultrasound-guided transbronchial needle aspiration(EBUS-TBNA), was performed to obtain the information of primary tumor and regional lymph nodes. Head involvement was examined by Magnetic Resonance Imaging(MRI).

**Drug treatment of neoadjuvant chemotherapy**

Overall, 21 patients(80.8%) with lung squamous cell carcinoma(LUSC), and 5 patients(41.7%) with lung adenocarcinoma(LUAD) were given albumin-bound paclitaxel or paclitaxel liposomes combined with platinum. Seven patients(58.3%) with LUAD and one patient(3.8%) with LUSC were treated with pemetrexed combined with platinum chemotherapy. Two patients(7.7%) with LUSC were given docetaxel plus platinum chemotherapy, and other two patients(7.7%) with LUSC received gemcitabine plus platinum chemotherapy.

**Examinations for clinical restaging**

Clinical restaging(ycStage) after neoadjuvant therapy was performed using imaging methods including CT, bone scan and/or PET-CT. Head involvement was examined by MRI.

**Surgical methods**

Surgical procedures include anatomical lobectomy(26, 68.4%), sleeve resection(9, 23.7%), or pneumonectomy(3, 7.9%), with ipsilateral and mediastinal hilar lymph node systematic dissection. At last, 35 patients(92.1%) achieved R0 resection.

**Supplementary Table S1. Details of included patients in this study.**

| Gender | Age | Pathological type | Driver mutation | Chemo-therapy cycles | ICI cycles | Cycle of neoadjuvant therapy | cStage | ycStage | Clinical response | Pathologic response | ypStage | Postchemo-  therapy | Postimmuno  -therapy | Recurrence  ~~Progression~~ | Survival |
| --- | --- | --- | --- | --- | --- | --- | --- | --- | --- | --- | --- | --- | --- | --- | --- |
| Male | 63 | LUSC | Not found | 3 | 2 | 5 | IIIA | IB | PR | non-MPR | IIB | No | Yes | Yes | Survive |
| Male | 75 | LUSC | Not found | 2 | 2 | 2 | IIIA | IIA | PR | MPR/pCR | pCR | Yes | Yes | No | Dead |
| Male | 66 | LUSC | Not found | 2 | 2 | 2 | IIIA | IIB | PR | non-MPR | IIB | No | Yes | Yes | Survive |
| Male | 53 | LUSC | Not found | 2 | 2 | 2 | IIIA | IB | PR | MPR/pCR | IIB | Yes | No | Yes | Dead |
| Male | 59 | LUSC | Not found | 2 | 2 | 4 | IIIA | IIIA | SD | MPR/pCR | pCR | No | Yes | No | Dead |
| Male | 57 | LUSC | Not found | 4 | 3 | 4 | IIIA | IIIA | SD | MPR/pCR | pCR | No | Yes | No | Dead |
| Male | 77 | LUSC | Not found | 2 | 3 | 3 | IIIA | IIB | PR | MPR/pCR | IA1 | Yes | Yes | No | Dead |
| Female | 62 | LUAD | Not found | 2 | 2 | 4 | IIIA | IIB | SD | non-MPR | IIIA | No | Yes | Yes | Survive |
| Male | 54 | LUSC | Not found | 2 | 2 | 2 | IIIA | IB | PR | non-MPR | IIB | Yes | Yes | Yes | Survive |
| Male | 72 | LUAD | Not found | 2 | 2 | 2 | IIIA | IIIA | SD | non-MPR | IIB | Yes | No | Yes | Survive |
| Male | 71 | LUSC | Not found | 2 | 2 | 2 | IIIA | IIA | SD | non-MPR | IIIA | Yes | No | Yes | Survive |
| Male | 65 | LUSC | Not found | 4 | 4 | 8 | IIIA | IIB | SD | non-MPR | IIIA | No | Yes | No | Dead |
| Male | 73 | LUSC | Not found | 3 | 3 | 3 | IIIA | IIIA | SD | MPR/pCR | IA2 | Yes | No | Yes | Dead |
| Male | 69 | LUSC | Not found | 2 | 2 | 2 | IIIA | IIA | PR | MPR/pCR | pCR | Yes | Yes | No | Dead |
| Male | 64 | LUAD | ERBB2-19 | 2 | 0 | 2 | IIIA | IB | PR | non-MPR | IIIA | Yes | No | Yes | Survive |
| Male | 70 | LUSC | Not found | 3 | 3 | 3 | IIIA | IIIA | SD | non-MPR | IIA | Yes | Yes | No | Dead |
| Male | 67 | LUSC | Not found | 2 | 2 | 2 | IIIA | IIB | PR | non-MPR | IA3 | Yes | No | No | Dead |
| Female | 56 | LUAD | Not found | 2 | 2 | 2 | IIIA | IIA | PR | non-MPR | IIB | Yes | Yes | No | Dead |
| Male | 75 | LUSC | Not found | 2 | 2 | 2 | IIIA | IIA | SD | non-MPR | IIIA | No | Yes | Yes | Survive |
| Male | 68 | LUSC | Not found | 2 | 2 | 2 | IIIA | IIB | PR | MPR/pCR | pCR | Yes | Yes | No | Dead |
| Male | 57 | LUSC | Not found | 4 | 4 | 4 | IIIA | IIB | PR | MPR/pCR | IA1 | Yes | No | No | Dead |
| Male | 78 | LUSC | Not found | 3 | 3 | 3 | IIIA | IIB | PR | non-MPR | IB | Yes | Yes | No | Dead |
| Male | 77 | LUSC | Not found | 2 | 2 | 2 | IIIA | IIA | PR | MPR/pCR | pCR | Yes | Yes | No | Dead |
| Male | 64 | LUSC | Not found | 3 | 0 | 3 | IIIA | IIB | PR | MPR/pCR | pCR | Yes | No | No | Dead |
| Male | 68 | LUAD | Not found | 4 | 4 | 4 | IIIA | IIA | PR | non-MPR | IA3 | No | Yes | No | Dead |
| Male | 54 | LUAD | EGFR-19 | 1 | 0 | 1 | IIIA | IIA | SD | non-MPR | IIIA | No | No | Yes | Dead |
| Male | 67 | LUSC | Not found | 4 | 4 | 4 | IIIA | IIIB | SD | MPR/pCR | pCR | No | Yes | Yes | Survive |
| Male | 61 | LUSC | Not found | 4 | 4 | 4 | IIIA | IIIB | SD | non-MPR | IVA | No | Yes | Yes | Survive |
| Female | 72 | LUAD | EGFR-19 | 2 | 0 | 2 | IIIA | IB | PR | non-MPR | IIB | Yes | No | Yes | Survive |
| Male | 67 | LUAD | Not found | 3 | 0 | 3 | IIIA | IIB | PR | non-MPR | IIB | Yes | Yes | Yes | Dead |
| Female | 64 | LUAD | Not found | 3 | 0 | 3 | IIIA | IIB | PR | non-MPR | IIB | No | No | No | Dead |
| Male | 73 | LUSC | Not found | 3 | 3 | 3 | IIIA | IIA | PR | non-MPR | IA3 | No | Yes | No | Dead |
| Male | 56 | LUSC | Not found | 2 | 2 | 2 | IIIA | IB | PR | MPR/pCR | pCR | No | Yes | No | Dead |
| Male | 70 | LUAD | EGFR-21 | 4 | 4 | 4 | IIIA | IIB | PR | non-MPR | IIB | Yes | Yes | Yes~~No~~ | Dead |
| Male | 54 | LUAD | KRAS | 2 | 2 | 2 | IIIA | IB | PR | MPR/pCR | pCR | No | Yes | No | Dead |
| Male | 67 | LUSC | Not found | 2 | 2 | 2 | IIIA | IIB | PR | MPR/pCR | pCR | No | Yes | No | Dead |
| Male | 72 | LUAD | Not found | 4 | 4 | 4 | IIIA | IIB | PR | MPR/pCR | pCR | No | Yes | No | Dead |
| Male | 69 | LUSC | Not found | 4 | 4 | 4 | IIIA | IIB | PR | non-MPR | IIB | No | Yes | Yes | Survive |

**ICI, immune checkpoint inhibitors; MPR, major pathologic response; pCR: pathological complete response; CR: complete response; PR: partial response; SD: stable disease; PD: progressive disease; NACT, neoadjuvant chemotherapy; NAICT, neoadjuvant immunochemotherapy; LUAD, lung adenocarcinoma; LUSC, lung squamous cell carcinoma.**

**
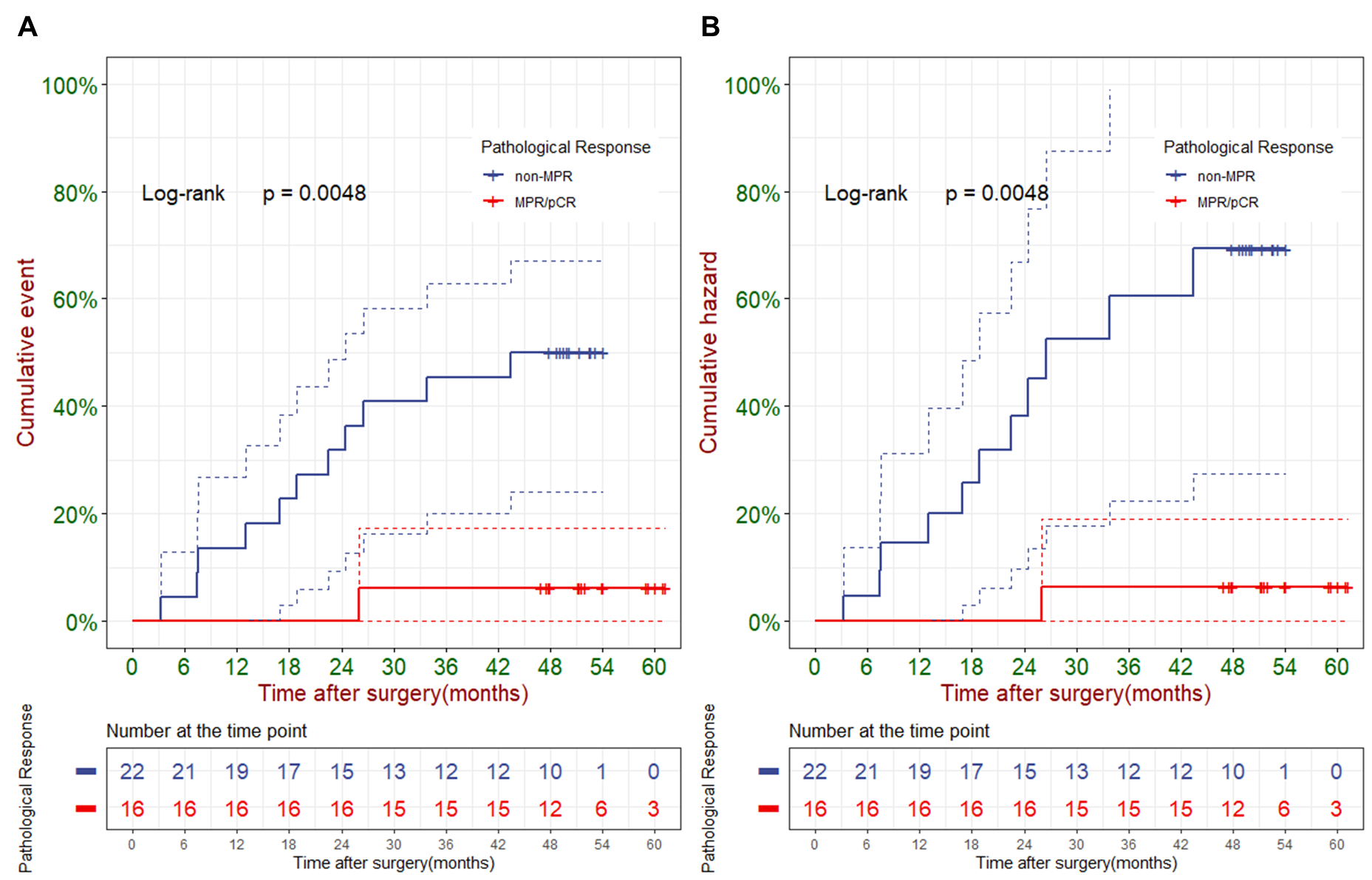
Supplementary Figure S1. Cumulative event and cumulative hazard curves of death among all patients according to their pathological response(A)(B).**

**
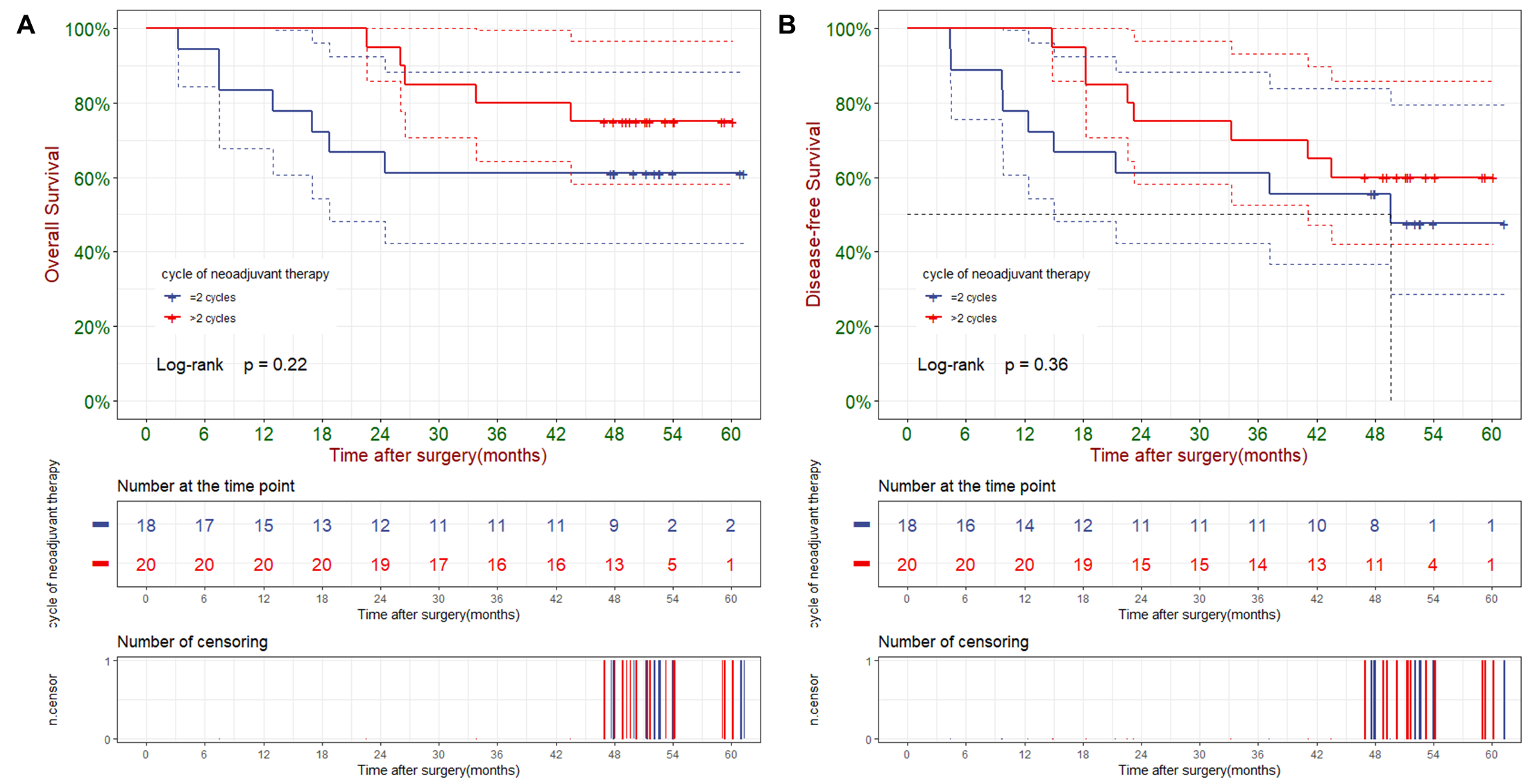
Supplementary Figure S2. Overall survival and disease-free survival of all patients according to their cycle of neoadjuvant therapy(A)(B).**
